# Characterization of Bacterial Microbiota Composition along the Gastrointestinal Tract in Rabbits

**DOI:** 10.3390/ani11010031

**Published:** 2020-12-26

**Authors:** Elisa Cotozzolo, Paola Cremonesi, Giulio Curone, Laura Menchetti, Federica Riva, Filippo Biscarini, Maria Laura Marongiu, Marta Castrica, Bianca Castiglioni, Dino Miraglia, Sebastiano Luridiana, Gabriele Brecchia

**Affiliations:** 1 Department of Agricultural, Food and Environmental Sciences, University of Perugia, Borgo XX Giugno 74, 06121 Perugia, Italy; elisa.cotozzolo@studenti.unipg.it; 2 Institute of Agricultural Biology and Biotechnology (IBBA)—National Research Council (CNR), U.O.S. di Lodi, Via Einstein, 26900 Lodi, Italy; cremonesi@ibba.cnr.it (P.C.); biscarini@ibba.cnr.it (F.B.); castiglioni@ibba.cnr.it (B.C.); 3 Department of Veterinary Medicine, University of Milano, Via dell’Università 6, 26900 Lodi, Italy; giulio.curone@unimi.it (G.C.); marta.castrica@unimi.it (M.C.); 4 Department of Agricultural and Food Sciences, University of Bologna, Viale G. Fanin 44, 40137 Bologna, Italy; laura.menchetti@unibo.it; 5 Department of Veterinary Medicine, University of Sassari, Via Vienna, 2, 07100 Sassari, Italy; marongiu@uniss.it (M.L.M.); sluridiana@uniss.it (S.L.); 6 Department of Veterinary Medicine, University of Perugia, Via San Costanzo 4, 06126 Perugia, Italy; dino.miraglia@unipg.it

**Keywords:** rabbit, intestinal microbiota, gastrointestinal tract, immune system, caecum

## Abstract

**Simple Summary:**

Each animal hosts a large community of bacteria, protozoa, fungi, and algae that colonize every surface of the body. The microbiota is defined as the complex microbial community that inhabits a specific anatomic region of animals and interacts with it, developing symbiotic relationships. In this regard, the intestinal microbiota has a vital impact on metabolism, digestive activity, immune development, resistance to infections, health, and welfare of the host. Therefore, knowing its characteristics is important for understanding its action in these specific functions. This study provides a comprehensive map of the most common bacterial communities that colonize different parts of the rabbit gastrointestinal tract (stomach, duodenum, jejunum, ileus, caecum, and colon) while using a modern methodological approach and comparing it with the studies that have been conducted to date on other animal species and human beings. It could be the starting point for further research on gut microbiota modulation with the ultimate goal to promote the health and welfare, in order to prevent or treat gastrointestinal diseases, decrease antibiotic use, and increase the productive performance of rabbit.

**Abstract:**

The microbiota is extremely important for the animal’s health, but, to date, knowledge on the intestinal microbiota of the rabbit is very limited. This study aimed to describe bacterial populations that inhabit the different gastrointestinal compartments of the rabbit: stomach, duodenum, jejunum, ileum, caecum, and colon. Samples of the luminal content from all compartments of 14 healthy New White Zealand rabbits were collected at slaughter and analyzed using next generation 16S rRNA Gene Sequencing. The findings uncovered considerable differences in the taxonomic levels among the regions of the digestive tract. Firmicutes were the most abundant phylum in all of the sections (45.9%), followed by Bacteroidetes in the large intestine (38.9%) and Euryarchaeota in the foregut (25.9%). Four clusters of bacterial populations were observed along the digestive system: (i) stomach, (ii) duodenum and jejunum, (iii) ileum, and (iv) large intestine. Caecum and colon showed the highest richness and diversity in bacterial species, while the highest variability was found in the upper digestive tract. Knowledge of the physiological microbiota of healthy rabbits could be important for preserving the health and welfare of the host as well as for finding strategies to manipulate the gut microbiota in order to also promote productive performance.

## 1. Introduction

The mammal’s gastrointestinal system is colonized by different microbial populations that live in a symbiotic relationship among them and with the host [1]. The characteristic microbial community that occupies a well-defined habitat and has distinct physio-chemical properties is defined as microbiota. The intestinal microbiota of the rabbit consists of microbial agents that belong to different kingdoms, such as bacteria, archea, protozoi, fungi, and algae, although bacteria are the most representative, with 100–1000 billions of microorganisms per gram and over 1000 different species [2,3]. This complex ecosystem plays important roles in various physiological processes of the host. In particular, the gut microbiota promotes the development and the maturation of the digestive system, contributes to food digestion, stimulates the immune system, and regulates the immune response; finally, it protects the host from pathogen colonization [4]. Vice versa, the host provides a suitable environment for the survival of microorganisms and their nutritional sustenance. In livestock animals, the bacterial communities play a key role in the maintenance of the health and welfare and, consequently, they can also influence their productive performance. Evidence in this regard has mainly concerned herbivorous species [5,6,7,8]. However, there are great differences in the structure and functions of the digestive tract among herbivores. Three categories can be identified: (i) ruminants, such as cattle and camelids, which have anterior digestive fermentative activity (rumen); (ii) posterior fermenters, such as horses and rodents, which have the large intestine (caecum and colon) as fermentation chamber; and, (iii) caecotrope animals, such as rabbits and hares, which rely on caecotrophy. 

Given that caecum is the primary site of fermentation, a large portion of studies on the microbiome of the digestive system of rabbit focused on the caecal and fecal bacterial communities [9,10,11,12,13,14]. The preliminary studies were animated to characterize the microbial population of caecum in order to understand its role in the digestive activity and health status of the rabbit [9,15,16,17]. Other studies investigated the bacterial communities that are involved in the improvement of health, welfare, and meat production of rabbits [18,19,20]. There are also pieces of evidence that fecal samples are quite similar with respect to the microbiome of large intestine, while their adequacy for other gastrointestinal tracts is still strongly doubtful [7,21].

Anyway, to date, only a few studies are available regarding the composition of the microbiota that inhabits the different sections of the gastrointestinal tract of healthy rabbits [22,23]. Hence, better knowledge and a complete microbial mapping of all different parts of the gastrointestinal tract are necessary for promoting the health and welfare of the rabbit, whose market is going through a period of difficulty and it is the subject of animal welfare issues [24,25]. Indeed, new strategies could be developed to favor the establishment of an advisable and “healthy” intestinal bacterial community that increases the digestible efficacy of the nutrient and, at the same time, saves energy for the host [17,26,27,28]. Moreover, the development of a beneficial bacterial population can promote the maturation and functioning of the immune system and, as a consequence, could reduce the incidence of infectious diseases, with beneficial effects on the health and welfare of animals. Finally, the characterization of the physiologic intestinal microflora found along a healthy gastrointestinal system of the rabbit could be helpful in defining new markers of health and pathological conditions for this species. Previous studies showed that several factors, including nutrition, genetics, and pathologies, can affect the productive and reproductive performance of rabbits and other livestock species acting through the microbiota [29,30,31,32,33,34,35]. 

This study investigated the microbial composition and diversity of the microbiota present in different tracts of the digestive system of adult rabbit (i.e., stomach, duodenum, jejunum, ileum, caecum, and colon) by 16S rRNA gene amplicon sequencing.

## 2. Materials and Methods 

### 2.1. Animals and Sample Collection

The trial was carried out at the experimental farm of the Department of Agricultural, Food, and Environmental Science of the University of Perugia. 

Fourteen New Zealand White Rabbits does were reared in single cages (dimensions: 38 cm W × 60 cm L × 35 cm H) and controlled environmental conditions: the temperature and relative humidity ranged between 18–21 °C and 60%, respectively; the artificial ventilation was 0.3 m3/s and the photoperiod of 16 h light per day at 40 lx. The animals were maintained in accordance with Legislative Decree No. 146, implementing Directive 98/58/EC regarding the protection of animals that were kept for farming purposes. The animals that were used in the study did not undergo any treatment or manipulation compared to the normal rabbit breeding system and, thus, authorization from the bioethical committee was not required. Moreover, the responsible veterinarian for the farm checked daily the rabbits for health and welfare states.

The rabbits were fed with standard pelleted feed (alfalfa meal 38 g/100 g, barley 19 g/100 g, maize gluten feed 15 g/100 g, extruded soybean 14 g/100 g, wheat bran 7 g/100 g, and vitamin and mineral mix 7 g/100 g) from weaning to 110 days of life. Throughout the entire trial, water and feed were provided ad libitum. The slaughter procedures are carried out in an authorized slaughterhouse and the stunning (electrical), bleeding, and skinning of the animals followed the European Union Regulations, specifically the Council Regulation No 1099/2009 on the protection of animals at the time of killing. At the slaughterhouse, the gastrointestinal tract was immediately removed. The content of stomach, duodenum, jejunum, ileum, colon, and caecum was collected in sterile tubes and then stored at −80 °C until the examination. 

### 2.2. Microbiota Evaluation 

#### 2.2.1. DNA Extraction

DNA was extracted from each fecal sample while using a QIAamp PowerFecal Pro DNA Kit (Qiagen, Hilden, Germany), according to the manufacturer’s protocol. DNA quality and quantity were assessed using a NanoDrop ND-1000 spectrophotometer (NanoDrop Technologies, Wilmington, DE, USA). The isolated DNA was then stored at −20 °C until use.

#### 2.2.2. 16S Ribosomal RNA (rRNA) Gene Sequencing 

Bacterial DNA was amplified while using the primers that were described in literature [36], which target the V3-V4 hypervariable regions of the 16S rRNA gene. All of the PCR amplifications were performed in 25 μL volumes per sample. A total of 12.5 μL of KAPA HIFI Master Mix 2× (Kapa Biosystems, Inc., Wilmington, MA, USA) and 0.2 μL of each primer (100 μM) were added to 2 μL of genomic DNA (5 ng/μL). The blank controls (no DNA template added to the reaction) were also performed. A first amplification step was performed in an Applied Biosystem 2700 thermal cycler (ThermoFisher Scientific, Waltham, MA USA). The samples were denatured at 95 °C for 3 min., followed by 25 cycles with a denaturing step at 98 °C for 30 s, annealing at 56 °C for 1 min. and extension at 72 °C for 1 min., with a final extension at 72 °C for 7 min. The amplicons were cleaned with Agencourt AMPure XP (Beckman, Coulter Brea, CA, USA) and libraries were prepared following the 16S Metagenomic Sequencing Library Preparation Protocol (Illumina, San Diego, CA, USA). The libraries obtained were quantified by Real Time PCR with KAPA Library Quantification Kits (Kapa Biosystems, Inc., Wilmington, MA, USA), pooled in equimolar proportion, and then sequenced in one MiSeq (Illumina, San Diego, CA, USA) run with 2 × 250-base paired-end reads. 

#### 2.2.3. Sequence Analysis

Demultiplexed paired-end reads from 16S rRNA-gene sequencing were first checked for quality while using FastQC [37] for an initial assessment. The forward and reverse paired-end reads were joined into single reads while using the C++ program SeqPrep [38]. After joining, the reads were filtered for quality based on: (i) maximum three consecutive low-quality base calls (Phred < 19) allowed; (ii) the fraction of consecutive high-quality base calls (Phred > 19) in a read over total read length ≥ 0.75; and, iii) no “N”-labeled bases (missing/uncalled) allowed. Reads that did not match all of the above criteria were filtered out. All of the remaining reads were combined in a single FASTA file for the identification and quantification of OTUs (operational taxonomic units). The reads were aligned against the SILVA closed reference sequence collection release 132, with 97% cluster identity [39,40] applying the Cd-hit clustering algorithm [41]. A pre-defined taxonomy map of reference sequences to taxonomies was then used for taxonomic identification along the main taxa ranks down to the genus level (domain, phylum, class, order, family, genus). By counting the abundance of each OTU, the OTU table was created and then grouped at each phylogenetic level. OTUs with total counts lower than 10 in fewer than two samples were filtered out. All of the above steps, except the FastQC reads quality check, were performed with the QIIME 1.9 open-source bioinformatics pipeline for microbiome analysis [42]. The command lines and parameters that were used to process 16S rRNA gene sequence data are detailed in Biscarini et al. [43].

#### 2.2.4. Alpha and Beta Diversity Indices

The microbial diversity of the different niches of the rabbit gastrointestinal tract was assessed within- (alpha diversity) and across- (beta diversity) samples. All of the indices (alpha and beta diversity) were estimated from the complete OTU table (at the OTU level), filtered for OTUs with more than 10 total counts distributed in at least two samples. Besides the number of observed OTUs directly counted from the OTU table, within-sample microbial richness, diversity, and evenness were estimated while using the following indices: Chao1 and ACE (Abundance-based coverage Estimator) for richness, Shannon, Simpson, and Fisher’s alpha for diversity [44,45,46,47,48,49], and Simpson E and Pielou’s J (Shannon’s evenness) for evenness [50]. The microbiota diversity across-sample was quantified by calculating Bray-Curtis dissimilarities [51]. Prior to the calculation of the Bray–Curtis dissimilarities, the OTU counts were normalized for uneven sequencing depth by cumulative sum scaling CSS, [52]. Details on the calculation of the mentioned alpha- and beta-diversity indices can be found in Biscarini et al. (S2 Appendix, [43]).

### 2.3. Statistical Analysis 

The rabbit’s core microbiota was identified by selecting OTUs that were shared by at least 95% of the samples. This was done over all the samples, and by anatomic region of the rabbit’s gastrointestinal tract. The function compute_core_microbiome.py from Qiime 1.9 was used. 

The analysis of variance (ANOVA) was used in order to test differences in alpha diversity indexes and OTU abundances—at various taxonomic levels along the rabbit’s gastrointestinal tract. For Bray–Curtis dissimilarities (beta diversity), differences along the digestive tract were tested non-parametrically while using the permutational analysis of the variance approach (999 permutations; [53]).

### 2.4. Software

Reads from 16S rRNA gene sequencing were processed with the QIIME 1.9 pipeline [42], used also to estimate most diversity indices. The ACE index and sample-base rarefaction were estimated while using own Python (https://github.com/filippob/Rare-OTUs-ACE.git) and R (https://github.com/filippob/sampleBasedRarefaction) scripts. The plots were generated while using the ggplot2 R package [54] (Wickham, 2009). Additional data handling and statistical analysis were performed with the R environment for statistical computing [55].

## 3. Results

### 3.1. Sequencing Metrics

The sequencing of the V3–V4 regions of the bacterial 16S rRNA gene produced a total of 8,235,038 reads (joined R1–R2 paired-end reads), with an average of 98,036 reads per sample (14 rabbits × 6 anatomical sections = 84 samples). After quality filtering, 1,885,925 sequences were removed, leaving 6,349,113 sequences for subsequent analyses (77% average retention rate, maximum 94%, minimum 56%). The average number of sequences was 75,584.7 (+/−38,864.1). Per anatomic section, the average number of sequences was 86,577.8 in the stomach, 97,499.7 in the duodenum, 67,446.7 in the jejunum, 71,851.2, in the ileum, 68,500 in the caecum, and 61,632.6 in the colon. All of the pairwise differences were not significant. 

**Figure 1:**
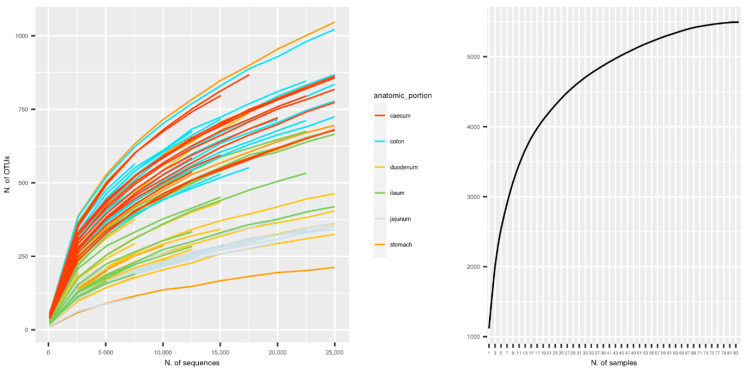
Rarefaction curves. Sequence-based (**left**) and sample-based (**right**) rarefaction curves for the sampled gastrointestinal tract microbiota. Number of detected Operational Taxonomic Units (OTUs) on the *y*-axis; number of sequences (**left**) and of samples (**right**) on the *x*-axis.

The initial number of OTUs identified was 5494; unique OTUs were unevenly distributed along the gastrointestinal tract, with a maximum of 2809 OTUs in the caecum and a minimum of 1624 OTUs in the jejunum. After pruning out OTUs with less than 10 counts in at least two samples, 1571 distinct OTUs were left. In order to check whether sequencing depth and sample size were adequate for characterizing the composition of the rabbit gut microbiota, sequence-based and sample-based rarefaction curves were generated from the OTU table before filtering (5494 OTUs). Sequence-based rarefaction curves were obtained from the QIIME pipeline (reference); the sample-based rarefaction curve was produced with ad hoc R functions. The observed number of OTUs detected was plotted as a function of the number of reads (up to 25,000) in each sample and of the number of specimens (Figure 1). Both of the curves tend to plateau asymptotically towards a maximum, indicating that sequencing depth, and the number of samples was adequate for characterizing the rabbit gut microbiota in the present study. Deeper sequencing or the addition of any other samples would not likely increase significantly the number of new OTUs discovered.

### 3.2. Taxonomic Characterization of Gut Microbiota Composition along the Gastrointestinal Tract

Table 1 shows the distribution of phylum relative abundances along the gastrointestinal tract. Firmicutes were consistently the most abundant phylum, accounting for around 40% of the gut microbiota everywhere, except the stomach, where they represented 68% of the microbiota composition. The second most abundant phylum was Bacteroidetes in the large intestine (caecum 39.6%, colon 38.2%) and in the stomach (15.9%); instead, in the small intestine, it was Euryarchaeota (jejunum 29.6%, duodenum 30.5%, ileum 17.6%). Additional phyla with sizable abundance included Verrucomicrobia (caecum 15.2%, colon 14.2%, and stomach 4.9%), Patescibacteria (jejunum 13.8%, duodenum 12.8%, ileum 10.1%), and Actinobacteria (jejunum 12.6%, duodenum 9.1%, ileum 8.9%, and the stomach 1.6%). 

**Figure 2:**
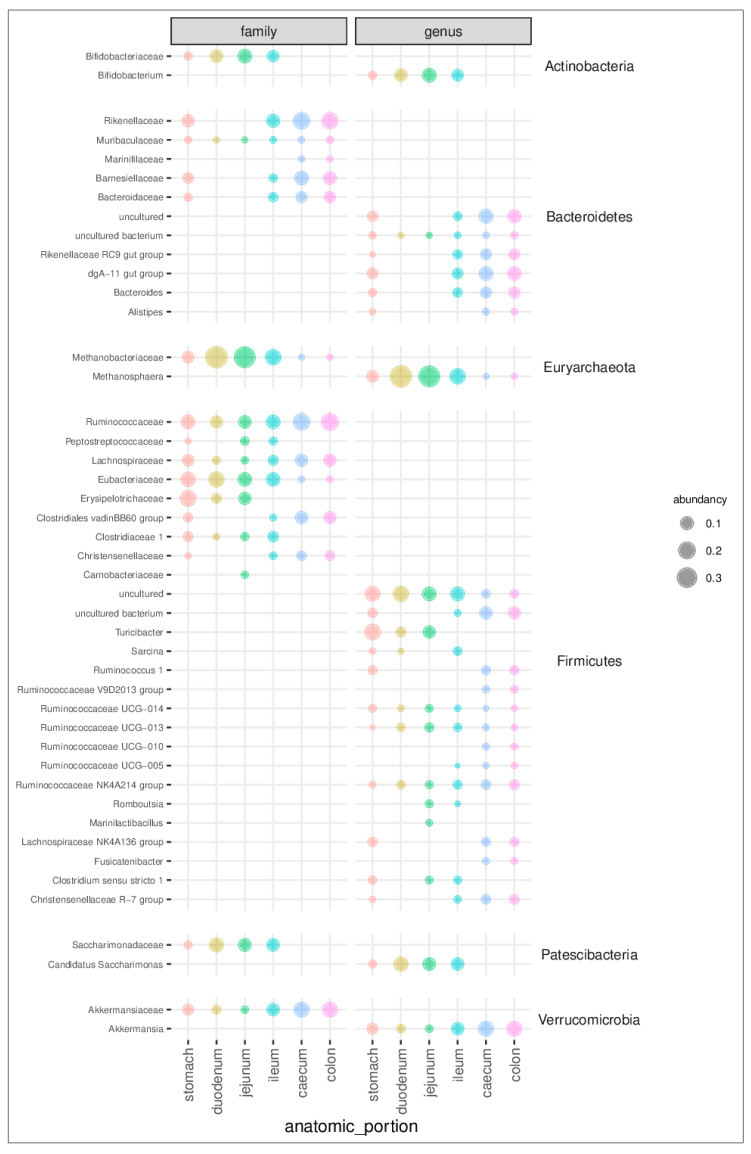
Bubble chart of relative abundances for the families and genera that were identified in the rabbit gut microbiota from 84 samples taken along the gastrointestinal tract (stomach, duodenum, jejunum, ileum, caecum, and colon). The names of the families and genera are reported on the left side of the figure. The name of phyla to which the different families and genera belong are reported on the right side of the figure. The central columns report the graphical representation of the abundance of the family and genus, respectively. Only taxa with relative abundance ≥ 1% are shown.

**Figure 3:**
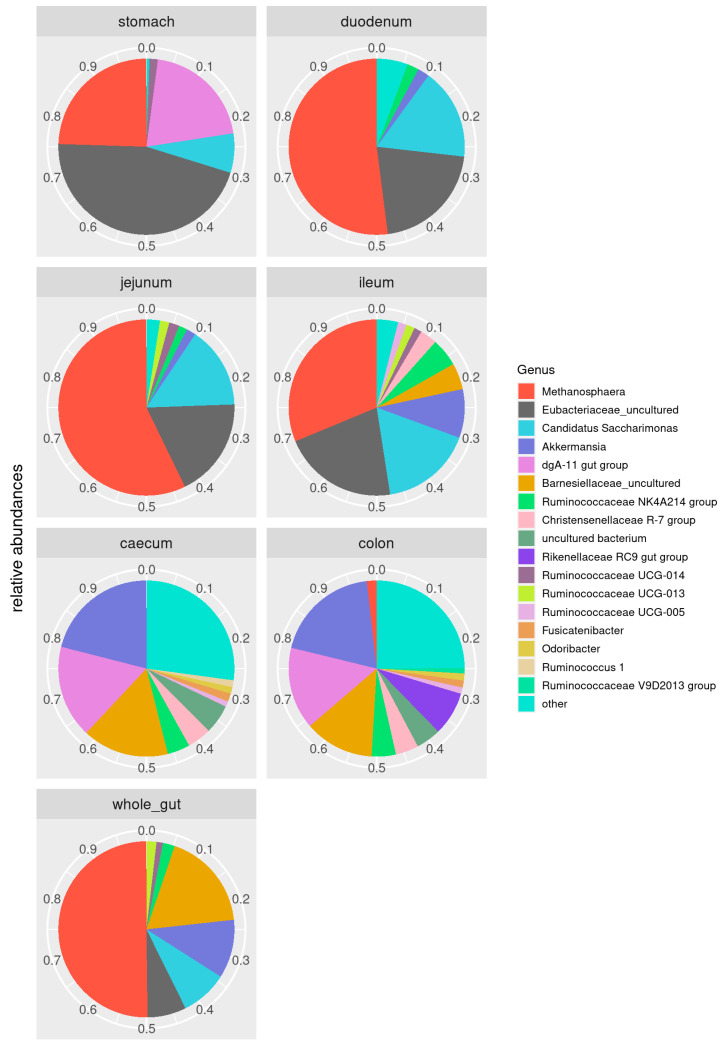
Core microbiota. Composition of the rabbit core gut microbiota (OTUs shared by at least 95% of the samples).

Figure 2 and Tables S1 and S2 report the relative abundance of bacterial families and genera per gastrointestinal tract. In the stomach, the most abundant families are Methanobacteriaceae (7%), Ruminococcaceae (12%), Rikenellaceae (8%), Eubacteriaceae (13%), Lachnospiraceae (7%), and Erysipelotrichaceae (17%). Methanobacteriaceae (38% and 35%), Eubacteriaceae (15% and 11%), Saccharimonadaceae (12% and 9%), Bifidobacteriaceae (9% and 11%), and Erysipelotrichaceae (4% and 8%) are the most abundant families in the duodenum and jejunum, respectively. The ileum shows a similar distribution, with the exception of Erysipelotrichaceae and the addition of Rikenellaceae (10%) and Akkermansiaceae (9%). Caecum and colon see a prevalence of Ruminococcaceae (19% and 20%), Rikenellaceae (19% and 18%), Akkermansiaceae (15% and 15%), Lachnospiraceae (8% and 8%), Barnesiellaceae (12% and 10%), Clostridiales vadinBB60 group (9% and 8%), and Bacteroidaceae (6% and 6%). At the genus level (Figure 3 and Table S2), Turicibacter dominates the stomach microbiota, where it represents 17.3% of the microbial community. Metanosphera (37.8% and 35.1%), Candidatus Saccharimonas (12.4% and 9.4%), and Bifidobacterium (8.9% and 11.7%) are the most abundant genera in the duodenum and jejunum. The ileum shows a similar distribution with fewer Metanosphera (15.9%) and more abundant Akkermansia (8.7%). Caecum and colon show a prevalence of Akkermansia (14.9% and 14.8%), dgA-11 gut group (11.7% and 10.9%), and uncultured Bacteroidetes (12.0% and 10.2%).

### 3.3. Core Microbiota

The core microbiome reflects those microbial taxa that are shared by 95% of the samples. We looked at the overall core microbiota (all samples, irrespective of the anatomic origin), and at the core mirobiota in each compartment of the gastrointestinal tract: the stomach, duodenum, jejunum, ileum, caecum, and colon core microbiota. Figure 3 shows the relative composition of the core microbiota along the gastrointestinal tract.

The bacterial taxa that are common across the entire rabbit gastrointestinal tract include the families Barnesiellaceae and Eubacteriaceae families, and the genera Metanosphera, Candidatus saccharimonas, Akkermansia genera, and three groups (NK4A214, UCG-013, and UCG-014) from the family Ruminococcaceae. In the caecum and colon, the core microbiome was dominated by the Rikenellaceae family and Akkermansia genus. Ileum, jejunum, and duodenum saw a prevalence of the Metanosphera and Candidatus Saccharimonas genera. In the ileum, the Metanosphera genus was less prevalent when compared to more proximal portions of the small intestine, with a comparatively larger representation of the genus Akkermansia. In the stomach, the most representative taxa of the core microbiota were the Metanosphera genus and the Rikenellaceae and Eubacteriaceae families. 

### 3.4. Alpha and Beta Diversity

**Figure 4:**
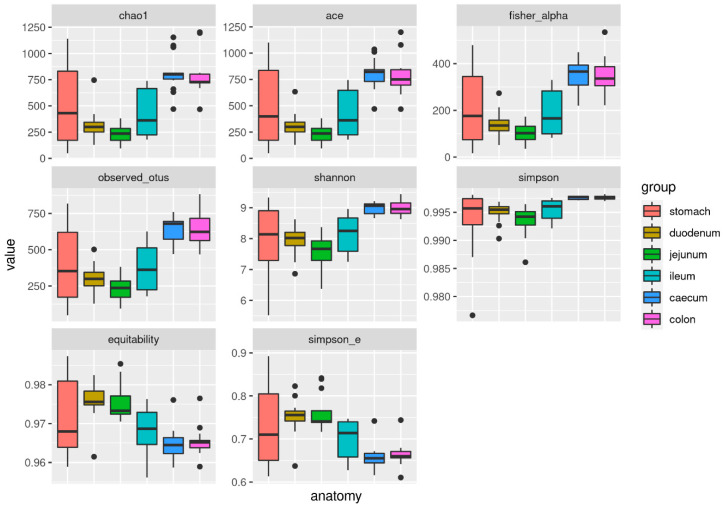
Alpha diversity indexes. Boxplots of alpha diversity indexes (Chao1, ACE, Fisher’s alpha, number of observed OTUs, Shannon diversity, Simpson diversity, equitability, Simpson E) along the different regions of the rabbit gastrointestinal tract.

The alpha diversity indexes that were measured in this study mainly show a divergence of caecum and colon on one hand, and duodenum and jejunum on the other, with the ileum and stomach appearing to generally occupy an intermediate position between them (Figure 4). 

From the analysis of variance, all of the indexes resulted in being significantly different along the gastrointestinal tract (null hypothesis: all of the indexes have the same value in all gut compartments). Taking caecum as the baseline, all of the other compartments except the colon had significantly different alpha diversity indexes (*p* < 0.001; Table 2).

**Figure 5:**
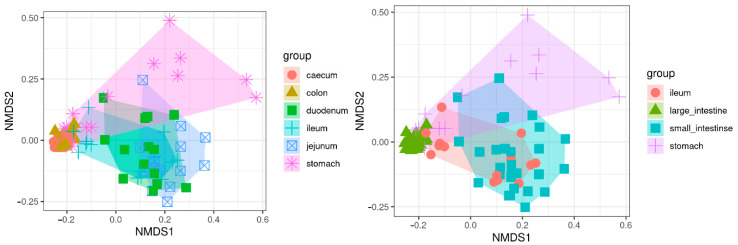
Beta diversity. Multidimensional scaling plot of Bray–Curtis distances between rabbit samples based on their microbiota. Left: clustering by the six regions of the gastrointestinal tract analyzed in this study. Right: regrouping based on observed microbiota similarities: large intestine (caecum and colon), small intestine (duodenum and jejunum), ileum and stomach. The first two dimensions from non-metric multidimensional scaling are plotted.

Beta diversity was measured as Bray–Curtis distances and revealed a similar pattern as alpha diversity with a clear clustering by compartment that emerges from the first two dimensions of the non-metric multidimensional scaling plot of between-sample distances (Figure 5). This clustering is more evident when grouping the compartments into large intestine, duodenum + jejunum (small intestine), and stomach, with the ileum close to the duodenum + jejunum group, but reaching out to the stomach and large intestine groups. The large intestine clearly forms a well-defined and compact cluster, indicating large similarities between the microbiota of the caecum and colon.

## 4. Discussion

The digestive tract of mammals is inhabited by an enormous number of symbiotic microorganisms that coexist and provide several ecosystem services, supplying functions that their host’s genome does not encode [56,57]. The knowledge of the physiologic gastrointestinal microbiota is crucial in promoting the growth, health, and welfare of the rabbit. Controlling the development of a beneficial commensal community, in fact, could favor functions of immune system and, consequently, the incidence of infectious diseases could be reduced. It could also result in the reduction of the use of antibiotics in the farm and then the development of antibiotic resistance. Moreover, a favorable intestinal microbiota can induce better digestive activity with a more efficient use of the diet, which, in turn, may increase the productive performance of the rabbits.

At present, only a few data on the gut microbiota of the rabbit are available in literature and the main information focused on the caecum and feces [3,12,58]. For this reason, this study aimed to investigate the spatial structure of the main bacterial populations of the rabbit gastrointestinal tract: stomach, duodenum, jejunum, ileum, caecum, and colon.

We detected twelve bacterial phyla along the gastrointestinal apparatus of the rabbit. In all of the digestive tracts analyzed, Firmicutes were largely the most abundant phylum and classified as the most efficient as cellulose degraders [58], so have a fundamental role in rabbits’ digestion. They ranged from 40% of the small and large intestinal segments to 68% of the stomach. Crowley et al. found a similar result in wild rabbits [22], although they found fewer and different phyla when compared to our findings (12 versus 8), probably as a consequence of the modern technique of assay utilized in the present study. Moreover, six out of the seven bacterial phyla identified in the Rex rabbits [16] were in common with our study. The differences in the composition of the microbiota could be explained by the anatomy, the environmental conditions (pH 1–2 in the stomach, around 7.2 and 6.5 in the small and large intestine, respectively, and aerobic level), and the different physiological functions of the various digestive compartments (tract length, interaction with the secretions, retention time, type digestion prevalent enzymatic or fermentative, etc.).

In agreement with our results, Firmicutes was the dominant phylum in both wild and Rex rabbits along all of the digestive system. Firmicutes resulted in being particularly abundant in the stomach of Rex and New Zealand White rabbits when compared to the wild rabbits (around 68.0% and 45.5%, respectively) [16,22]. However, a large part of Firmicutes of Rex rabbits in foregut was dead, and the most abundant phylum of live bacteria was instead the Proteobacteria. In fact, a higher percentage of dead bacteria was found in the first tracts of the digestive system, with respect to caecum and colon, probably as a consequence of the gastric acid environment [16]. This evidence suggests that dead bacteria might interfere with the bacterial count in particular in the stomach and foregut, and especially in cecotrope animals, which introduce an external surge of bacteria. In fact, in the Rex rabbit, the profile of the large intestine remained substantially unaltered when considering the total and live bacteria counts [16]. Firmicutes represent the dominant phylum in almost all of the digestive tracts also in other monogastric species: herbivorous, omnivorous, broilers, and humans. The sequence abundance of the bacteria that belong to these phyla is greater in rabbit respect to broiler and pig, very similar to those of horse, and lower in comparison to humans [21,59,60,61]. In our study, we observed a drastic abundance reduction passing from the stomach to duodenum, which is mainly due to the slump of a single family/genus of bacteria. Indeed, Turicibacter represents 17.0% of Firmicutes in the stomach and 2.5% in the intestine. The presence at high levels of Turicibacter in the stomach was also described in laboratory rats [62]. Very little is known regarding Turicibacter species and their biological relevance in physiology and pathology. *T. sanguinis* is the only species described of this family and it was detected in human patients that are affected by appendicitis and ulcerative colitis [63,64,65], in mice [66], and in pigs [67].

Bacteroidetes was the second abundant phylum in the gastrointestinal tract of rabbits in our study. Bacteroidetes are one of the major commensal phyla in the gut of rabbits and they were proven to stimulate the development of gut-associated immune tissue [12,58]. Their sequences were particularly high in the hindgut (around 40%), showed intermediate value in stomach and ileum (around 16%), and they were very low in duodenum and jejunum. Similar results were found in large intestine segments of wild rabbits and Rex rabbit [16,22].

Our results showed that the Proteobacteria was evidenced at very low sequence levels in all of the gastrointestinal tracts; on the contrary, higher quantity have been found in Rex and wild rabbits, as well as in broiler [61], horse [21,68], pig [59], and human [60].

In the small intestine of the rabbit, there were quite abundant levels of bacteria belonging to the phylum Euryarchaeota (methanogenic producing bacteria), representing the largest phyla after the Firmicutes in this site. Partially in agreement with our findings, Euryarchaeota are present in Rex rabbit in the same intestinal sections, but in less quantity [16], while, in wild rabbits, this phylum was not detected [22]. Euryarchaeota is not very common in the intestinal microflora of horses [21,68,69], humans [70], and pig [71].

In our study, the phylum Verrucomicrobia was detected with sizable abundance in all the digestive tract, but, in particular, in large intestine, while it has not been identified in Rex or wild rabbits [16,22]. With respect to our results, Verrucomicrobia was more abundant in the large bowel of the horse [21,68,69] and it showed lower levels in the colon of humans [60] as well as in the duodenum and colon of pigs [71].

Patescibacteria is a phylum detected in discrete quantity in the small intestine of our animals, but, conversely, it has not been found in wild and Rex rabbits as well as in other animal species.

In agreement with the Rex rabbit’s study, we found an abundant concentration of bacteria that belong to Actinobacteria in the small intestine of New White Zealand rabbits. It was observed at very low sequence levels in the wild rabbit [22], while it was detected in all of the gastrointestinal tract of broiler and pig [61,71]. The other bacterial phyla found in the different digestive tracts had very low concentrations in all the segments analyzed.

In our study, four different clusters of the gastrointestinal microbiota were identified: stomach, small intestine (duodenum and jejunum), ileum, and large intestine (caecum and colon). The ileum showed a commensal population that is similar to small and large intestine, given that it receives nutrients and microbial agents through the peristaltic movements by the hindgut as well as the reflux of materials coming back from the caecum. Our results indicated that the gastrointestinal microbial community varied widely between some sections of the digestive system in rabbits, but these variations were less evident in adjacent tracts. This observation is also confirmed by the β-diversity analysis. In general, an increased diversity in bacterial composition towards the foregut and the large intestine was found in New White Zealand rabbits. In fact, the hindgut shows a higher complexity, richness, and diversity in bacterial species, which was probably linked to its the fermentative function and caecotrophy, with respect to the stomach and small intestine. These expected results could reflect the co-evolution of microbiota and the digestive tract of the host, which is composed of several macroniches that are characterized by different physicochemical conditions. These specific conditions force bacteria to adapt and represent an environmental selective pressure factor. In Rex rabbits and horses, two clusters were identify for the whole digestive system (foregut and large intestine) [16,21], while three clusters were found in broiler [61]. Hence, it could be speculated that the high diversity in the microbiota of the upper gastrointestinal tract of different animal species could be linked to the assumption of environmental bacterial with the introduction of feed, forage, and grooming, and also by caecotrophy for the rabbit. Conversely, the large intestine is remarkably stable and uniform, because it is less influenced by environmental bacterial.

It is important highlight that the knowledge concerning the families and genera of the microbiota in the different tracts of the digestive system of the rabbit is very limited, with the exception of the caecum. The analysis of the genera and families confirmed the clustering in four groups of the digestive apparatus of the New White Zealand rabbits, as reported for the phyla.

In the stomach of New White Zealand rabbits, we found that the most relative abundant families were Erysipelotrichaceae, Eubacteriaceae, Ruminococcaceae, Methanobacteriaceae, and Lachnospiraceae. In the same compartment, at the genus level, we found as dominant bacteria Turicibacter, Methanosphaera, Akkermansia, Ruminococcus 1, and Ruminococcaceae UCG-014, while, in Rex rabbits, were observed Acinetobacter, Cupriavidus, Clostridiales and Ruminococcaceae in live bacteria group [16]. In the small intestine, the most abundant families found on New White Zealand rabbits were Methanobacteriacea, Eubacteriaceae, Saccharimonadaceae, Bifidobacteriaceae, and Erysipelotrichaceae. Producing methane bacteria belonging Methanosphaera are the dominant genus in the first two segments of the intestine, followed by Bifidobacterium, Turibacter, and the different genus of ruminococcaceae. Acinetobacters, Cupriavidus, Clostridiales, Ruminococcaceae, and Halomonas showed the highest sequences in the jejunum of Rex rabbits [16]. Ileum showed a similar distribution in families and genera in comparison to duodenum and jejunum in New White Zealand rabbits, although the appearance of Bacteroides, Rikenellaceae RC9 gut group, and Christensenellaceae R7 group was observed. In Rex rabbit, Fu et al. [16] found the same genera of jejunum, although with different abundance.

The abundance of the bacterial families changed significantly in the caecum and colon of New White Zealand rabbits. Methanobacteriaceae and Eubacteriaceae families were strongly reduced and Erysipelotrichaceae was not detected in large intestine. Ruminococcaceae, Akkermansiaceae, and Lachnospiraceae increased their quantity with respect to the other intestinal tracts. Finally, unlike small intestine, Rikenellaceae, Barnesiellacea, Clostridiales, Bacteroidacea, and Christensenellaceae were detected in caecum and colon. Concerning the bacterial genera, the most abundant were Akkermansia, Bacteroides, and some genera belonging to Ruminococcaceae and Ruminococcus, Rikenellaceae RC9 gut group, Christensenellaceae R7 group, and Fusicatenibacter. In the Rex rabbit, the authors found that the Clostridiales, Ruminococcaceae, and S24-7 were the dominant genera [16]. Especially, Ruminococcaceae and Lachnospiraceae are considered to be important indexes of intestinal health; higher values of Lachnospiraceae are characteristic of healthy rabbits, due to the stimulation of caecotrophic behavior, which was also associated with a reduction in the mortality in young rabbits. Ruminococcus is the most relevant genus of the Firmicutes phylum that is dominant in healthy rabbits and it decreases in the presence of disease [72].

The microbial population of the caecum is that most abundantly studied in rabbit, given that it represents the most important site of microbial fermentation of indigestible dietary fibers and the production of short-chain fatty acids, which are involved in the regulation of energy metabolism as well as in the maintaining of the intestinal homeostasis [73,74]. In agreement with our results, several studies found Firmicutes to be the most representative phylum of the caecum microbiome, but with some differences in relative abundance percentages and Firmicutes and Bacteroidetes ratio [11,18,33]. The Firmicutes and Bacteroidetes ratio could be evaluated as a marker of dysbiosis in rabbits, likewise in humans [75] and horse [21].

## 5. Conclusions

In conclusion, our findings, despite the limitation of the number of animals used in the trial, lead to a better understanding of the microbiota of the different tracts of the digestive system of the New White Zealand rabbit. Moreover, we found differences in the microbial community along the various tracts of the gastrointestinal system that could be divided into four compartments: stomach, small intestine, ileum, and large intestine. Besides this, an increment of the stability of the bacterial communities from the stomach to colon was also relieved. Future studies should be addressed in order to increase the number of intestinal microbiota sequenced in order to confirm our findings and evaluate the effect of different factors, such as the genetic, diet, management techniques, drugs, and pathologies on the shift of the intestinal microbial population and, as a consequence, on the health and the production of the rabbit.

## Figures and Tables

**Table 1:** Relative abundance of Operational Taxonomic Units (OTUs) of phyla that are significantly different between the six regions of the gastrointestinal tract analyzed in this study (stomach, duodenum, jejunum, ileum, caecum, and colon). Values are means ± standard deviations. The cut-off value for significance was *p*-value < 0.01.

Phylum	Stomach	Duodenum	Jejunum	Ileum	Caecum	Colon	*p*-Value
Firmicutes	0.68 ± 0.27	0.40 ± 0.23	0.41 ± 0.26	0.40 ± 0.19	0.43 ± 0.08	0.44 ± 0.07	0.00
Bacteroidetes	0.16 ± 0.16	0.02 ± 0.03	0.01 ± 0.02	0.16 ± 0.16	0.40 ± 0.06	0.38 ± 0.08	0.00
Verrucomicrobia	0.05 ± 0.05	0.04 ± 0.04	0.02 ± 0.02	0.08 ± 0.07	0.15 ± 0.04	0.14 ± 0.03	0.00
Patescibacteria	0.03 ± 0.02	0.13 ± 0.11	0.14 ± 0.13	0.10 ± 0.11	0.01 ± 0.00	0.01 ± 0.01	0.00
Euryarchaeota	0.06 ± 0.09	0.31 ± 0.26	0.30 ± 0.28	0.18 ± 0.23	0.01 ± 0.01	0.02 ± 0.02	0.00
Actinobacteria	0.02 ± 0.03	0.09 ± 0.16	0.13 ± 0.22	0.09 ± 0.18	0.00 ± 0.01	0.00 ± 0.00	0.06
Cyanobacteria	0.01 ± 0.01	0.01 ± 0.01	0.00 ± 0.01	0.00 ± 0.00	0.00 ± 0.00	0.00 ± 0.00	0.14
Proteobacteria	0.00 ± 0.00	0.00 ± 0.00	0.00 ± 0.00	0.00 ± 0.00	0.00 ± 0.00	0.00 ± 0.00	0.02
Tenericutes	0.00 ± 0.00	0.00 ± 0.00	0.00 ± 0.00	0.00 ± 0.00	0.00 ± 0.00	0.00 ± 0.00	0.59
Epsilonbacteraeota	0.00 ± 0.00	0.00 ± 0.01	0.00 ± 0.00	0.00 ± 0.00	0.00 ± 0.00	0.00 ± 0.00	0.13
Lentisphaerae	0.00 ± 0.00	0.00 ± 0.00	0.00 ± 0.00	0.00 ± 0.00	0.00 ± 0.00	0.00 ± 0.00	0.85
Synergistetes	0.00 ± 0.00	0.00 ± 0.00	0.00 ± 0.00	0.00 ± 0.00	0.00 ± 0.00	0.00 ± 0.00	0.00

**Table 2:** Significance of the difference in alpha diversity indexes between regions of the rabbit’s gastrointestinal tract. Benchmark is the caecum microbiota alpha diversity. Values are means ± standard deviations.

Metric	Stomach	Duodenum	Jejunum	Ileum	Caecum	Colon	*p*-Value
Chao1	508.51 ± 389.20	316.86 ± 148.15	233.50 ± 85.33	432.19 ± 220.60	814.53 ± 181.13	787.36 ± 192.90	0.00
Ace	493.36 ± 376.40	308.82 ± 124.18	233.50 ± 85.33	424.02 ± 214.95	803.85 ± 147.50	785.11 ± 181.83	0.00
Fisher_alpha	210.88 ± 163.59	139.61 ± 57.78	102.52 ± 40.79	187.01 ± 94.71	354.15 ± 60.28	348.24 ± 78.62	0.00
Observed otus	399.71 ± 271.22	299.43 ± 100.43	233.50 ± 85.33	374.86 ± 160.72	645.43 ± 84.73	638.79 ± 111.72	0.00
Shannon	7.97 ± 1.17	7.94 ± 0.50	7.58 ± 0.56	8.15 ± 0.60	8.99 ± 0.18	8.98 ± 0.23	0.00
Simpson	0.99 ± 0.01	1.00 ± 0.00	0.99 ± 0.00	1.00 ± 0.00	1.00 ± 0.00	1.00 ± 0.00	0.00
Equitability	0.97 ± 0.01	0.98 ± 0.00	0.98 ± 0.00	0.97 ± 0.01	0.96 ± 0.00	0.97 ± 0.00	0.00
Simpson e	0.73 ± 0.10	0.75 ± 0.04	0.76 ± 0.04	0.70 ± 0.04	0.66 ± 0.03	0.66 ± 0.03	0.00

## Supplementary Materials

The following are available online at https://www.mdpi.com/2076-2615/11/1/31/s1, Table S1: Relative abundances of microbial families in the rabbit gut microbiome, along the different compartments of the gastrointestinal tract, Table S2: Relative abundances of microbial genera in the rabbit gut microbiome, along the different compartments of the gastrointestinal tract.
