# Supplementary material for: Characterization of Bacterial Microbiota Composition along the Gastrointestinal Tract in Rabbits
Source: Animals (Basel). 2020 Dec 26;11(1):31. doi: 10.3390/ani11010031 (PMC7824689; doi:10.3390/ani11010031)
Supplement: Supplementary file 1 [file animals-11-00031-s001.pdf]

animals

MDPI

Supplementary materials

Table S1. Relative abundances of microbial families in the rabbit gut microbiome, along the different compartments of the gastrointestinal tract. P-values come from analysis of variance (H0: relative abundances are equal in all compartments).

Phylum	Taxa: Family	Stomach	Duodenum	Jejunum	Ileum	Caecum	Colon	P value
Actinobacteria	Bifidobacteriaceae	0.025	0.089	0.117	0.071	0.000	0.000	0.291
Bacteroidetes	Bacteroidaceae	0.026	0.000	0.000	0.037	0.063	0.064	0.001
Bacteroidetes	Barnesiellaceae	0.052	0.000	0.000	0.026	0.117	0.098	0.000
Bacteroidetes	Marinifilaceae	0.000	0.000	0.000	0.000	0.013	0.014	0.000
Bacteroidetes	Muribaculaceae	0.019	0.013	0.012	0.015	0.014	0.019	0.774
Bacteroidetes	Rikenellaceae	0.084	0.000	0.000	0.100	0.191	0.185	0.000
Euryarchaeota	Methanobacteriaceae	0.072	0.379	0.352	0.159	0.012	0.013	0.002
Firmicutes	Carnobacteriaceae	0.000	0.000	0.019	0.000	0.000	0.000	0.462
Firmicutes	Christensenellaceae	0.014	0.000	0.000	0.021	0.038	0.041	0.000
Firmicutes	Clostridiaceae 1	0.044	0.016	0.027	0.054	0.000	0.000	0.318
Firmicutes	Clostridiales vadinbb60 group	0.038	0.000	0.000	0.016	0.086	0.080	0.000
Firmicutes	Erysipelotrichaceae	0.173	0.043	0.085	0.000	0.000	0.000	0.601
Firmicutes	Eubacteriaceae	0.134	0.155	0.113	0.108	0.014	0.015	0.000
Firmicutes	Lachnospiraceae	0.074	0.023	0.019	0.046	0.084	0.083	0.000
Firmicutes	Peptostreptococcaceae	0.012	0.000	0.029	0.024	0.000	0.000	0.769
Firmicutes	Ruminococcaceae	0.122	0.083	0.089	0.115	0.192	0.205	0.000
Patescibacteria	Saccharimonadaceae	0.025	0.124	0.094	0.088	0.000	0.000	0.000
Verrucomicrobia	Akkermansiaceae	0.061	0.031	0.021	0.087	0.149	0.148	0.000

Table S2. Relative abundances of microbial genera in the rabbit gut microbiome, along the different compartments of the gastrointestinal tract. P-values come from analysis of variance (H0: relative abundances are equal in all compartments).

Phylum	Taxa: Genus	Stomach	Duodenum	Jejunum	Ileum	Caecum	Colon	P value
Actinobacteria	Bifidobacterium	0.025	0.089	0.117	0.071	0.000	0.000	0.291
Bacteroidetes	Alistipes	0.013	0.000	0.000	0.000	0.016	0.016	0.000
Bacteroidetes	Bacteroides	0.026	0.000	0.000	0.037	0.063	0.064	0.001
Bacteroidetes	Dga-11 gut group	0.060	0.000	0.000	0.056	0.117	0.110	0.000
Bacteroidetes	Rikenellaceae rc9 gut group	0.011	0.000	0.000	0.037	0.058	0.058	0.000
Bacteroidetes	Uncultured	0.052	0.000	0.000	0.029	0.120	0.102	0.441
Bacteroidetes	Uncultured bacterium	0.019	0.012	0.012	0.015	0.014	0.018	0.196
Euryarchaeota	Methanosphaera	0.072	0.379	0.351	0.159	0.012	0.013	0.002
Firmicutes	Christensenellaceae r-7 group	0.014	0.000	0.000	0.021	0.038	0.041	0.000
Firmicutes	Clostridium sensu stricto 1	0.027	0.000	0.022	0.024	0.000	0.000	0.681
Firmicutes	Fusicatenibacter	0.000	0.000	0.000	0.000	0.017	0.015	0.001
Firmicutes	Lachnospiraceae nk4a136 group	0.039	0.000	0.000	0.000	0.031	0.032	0.000
Firmicutes	Marinilactibacillus	0.000	0.000	0.016	0.000	0.000	0.000	0.469
Firmicutes	Romboutsia	0.000	0.000	0.024	0.011	0.000	0.000	0.761
Firmicutes	Ruminococcaceae nk4a214 group	0.017	0.027	0.024	0.033	0.040	0.046	0.000
Firmicutes	Ruminococcaceae ucg-005	0.000	0.000	0.000	0.010	0.013	0.015	0.000
Firmicutes	Ruminococcaceae ucg-010	0.000	0.000	0.000	0.000	0.018	0.017	0.000
Firmicutes	Ruminococcaceae ucg-013	0.010	0.025	0.034	0.026	0.014	0.014	0.377
Firmicutes	Ruminococcaceae ucg-014	0.024	0.015	0.022	0.014	0.011	0.014	0.447
Firmicutes	Ruminococcaceae v9d2013 group	0.000	0.000	0.000	0.000	0.019	0.021	0.000
Firmicutes	Ruminococcus 1	0.033	0.000	0.000	0.000	0.035	0.035	0.000
Firmicutes	Sarcina	0.016	0.012	0.000	0.030	0.000	0.000	0.511
Firmicutes	Turicibacter	0.173	0.043	0.085	0.000	0.000	0.000	0.600
Firmicutes	Uncultured	0.145	0.158	0.115	0.115	0.028	0.030	0.441

**Firmicutes**	**Uncultured bacterium**	0.039	0.000	0.000	0.016	0.086	0.080	0.196
**Patescibacteria**	**Candidatus saccharimonas**	0.025	0.124	0.094	0.088	0.000	0.000	0.000
**Verrucomicrobia**	**Akkermansia**	0.061	0.031	0.021	0.087	0.149	0.148	0.000
